# The Basic Act for Suicide Prevention: Effects on Longitudinal Trend in Deliberate Self-Harm with Reference to National Suicide Data for 1996–2014

**DOI:** 10.3390/ijerph14010104

**Published:** 2017-01-21

**Authors:** Miharu Nakanishi, Kaori Endo, Shuntaro Ando

**Affiliations:** 1 Mental Health and Nursing Research Team, Tokyo Metropolitan Institute of Medical Science, 2-1-6 Kamikitazawa, Setagaya-ku, Tokyo 156-8506, Japan; 2 Mental Health Promotion Project, Tokyo Metropolitan Institute of Medical Science, 2-1-6 Kamikitazawa, Setagaya-ku, Tokyo 156-8506, Japan; endo-kr@igakuken.or.jp (K.E.); ando-st@igakuken.or.jp (S.A.)

**Keywords:** deliberate self-harm, health policy, suicide prevention

## Abstract

A suicide prevention strategy was launched in Japan in 2006 to address the high suicide rate, which had increased considerably since 1998. The national strategy from 2007 involved the enhancement of psychiatric treatment services at emergency medical facilities and supportive observation by individuals close to patients. The national suicide rate has decreased gradually since 2008; however, national information regarding the number of patients who had engaged in deliberate self-harm was absent. Therefore, the present study examined the longitudinal trend in hospital admissions due to deliberate self-harm in Japan. Data from the National Patient Survey between 1996 and 2014—a nationally representative cross-sectional survey of inpatient care every 3 years—were used. Data for 13,014 patients were included in the estimation of the number of hospital admissions due to deliberate self-harm. The results show that the estimated number of admissions due to deliberate self-harm increased from 2078 in September 1996 to 3189 in September 2008, when the national number of suicide cases peaked, and decreased to 1783 in 2014. Approximately half of the patients were admitted to hospital because of self-harm via means other than drug poisoning, which had a high mortality rate (5.6%). The proportion of patients receiving public assistance was higher in those who had engaged in deliberate self-harm (8.5%) relative to that observed in the general population. Overall, the trend in deliberate self-harm was synchronous with the number of suicide cases over time. As economic poverty has been associated with suicidal ideation and behavior and some recipients of public assistance tend to abuse psychotropic medication, the public assistance program should provide mental health support for recipients of social benefit schemes.

## 1. Introduction

Suicide is a major global public health problem. It has been estimated that over 800,000 people die by suicide annually, with numerous suicide attempts made prior to death [1]. Previous deliberate acts of self-harm is a key risk factor for suicide [2,3,4,5]. Although deliberate self-harm has been defined as intentional self-poisoning or self-injury regardless of the underlying motivation and of any intent to die [6], there is overlap between deliberate self-harm and suicidal intentions [7]. There is a distinction between medically serious self-harm that requires treatment in a hospital and other forms of self-harm, which do not require medical attention and might only be included in community samples. Admission to a hospital in the former population can be a cue as to the need for intervention. Interventions involving active contact and follow-up are effective in preventing further suicidal behavior in patients admitted to emergency departments following suicide attempts [8]. However, no reports regarding the national prevalence of deliberate self-harm have been produced; therefore, little attention has been paid to such care. Emergency treatment for patients who engage in deliberate self-harm sometimes lacks services such as mental health assessment [9] and positive encounters [10].

Japan’s suicide rate is higher relative to those of other Organization for Economic Co-operation and Development countries, and increased considerably following the 1998 economic recession [11]. In 2006, the Basic Act for Suicide Prevention allocated responsibility for suicide prevention to all stakeholders—including municipal governments—in Japan [12]. In 2007, the Japanese Cabinet Office released the General Principles of Suicide Prevention Policy; this included an initiative concerning “Efforts to Prevent Attempted Suicide Victims from Reattempting Suicide”, which involved the enhancement of psychiatric treatment services at emergency medical facilities and support for observation by individuals close to patients (family members) [13]. Economic recession has been found to exert a negative impact on self-harm and suicide [14]; therefore, the longitudinal trend in deliberate self-harm requires examination. However, information regarding the national number of hospital admissions due to deliberate self-harm is scarce in Japan. Consequently, the effect of the national suicide prevention strategy on deliberate self-harm rates has not been examined. Therefore, the present study aimed to examine the prevalence of and time trend in hospital admissions due to deliberate self-harm in Japan. In relation to economic recession, we investigated the association between methods of self-harm behavior and the proportion of public assistance.

## 2. Materials and Methods

### 2.1. Design

The study used a cross-sectional retrospective design that sampled data from the National Patient Survey—a nationally representative cross-sectional survey of hospital services provided under the public healthcare insurance program, conducted in Japan. A detailed description of the National Patient Survey has been reported elsewhere [15].

### 2.2. Setting

The National Patient Survey is conducted by the Ministry of Health, Labour, and Welfare in October every 3 years, and includes inpatient surveys. The current study used discharge information from databases for 1996–2014, as this included the economic recession (1997) and introduction of the Basic Act for Suicide Prevention (2006). Permission to access the National Patient Survey data during the study period was obtained from the Ministry of Health, Labour, and Welfare.

The National Patient Survey used a two-stage stratified random sampling procedure. In the first stage, 76% of all hospitals were selected; in the second stage, discharge samples were selected from the chosen hospitals. The managing director of each hospital was asked to provide patient data on a designated date between 21 and 23 October every 3 years. Patient data were examined for 1 to 30 September every 3 years between 1996 and 2014.

### 2.3. Participants

In total, 5,978,024 patient-level questionnaires were collected during the study period. Data were extracted for 13,014 patients who had engaged in deliberate self-harm, and whose primary disease code fell between S00 and T98 in the International Statistical Classification of Diseases and Related Health Problems, 10th Revision (ICD-10). Data for these patients were included in the estimation of the national number of hospital admissions due to deliberate self-harm.

### 2.4. Measurements

The National Patient Survey includes questions pertaining to discharged patients’ primary diseases, deliberate self-harm, treatment outcomes, and characteristics. A weighting coefficient was allocated to each patient based on the sampling rate. Regional residence and hospital characteristics were derived from the Survey of Medical Institutions.

Primary diseases were classified using ICD-10 codes A00–T98. When the primary disease was coded as injury, poisoning, and certain other consequences of external causes (S00–T98), external causes were classified into four categories: deliberate self-harm, harm caused by others, accident, and other/not specified. The presence of deliberate self-harm was used to extract data for the patient sample. Based on previous studies that reported fatalities occurring due to different means of self-harm [2,3,5], patients were classified into the following three categories: drug poisoning, substance poisoning, and other self-injury.

Treatment outcomes were classified into six categories: cured, improved, unchanged, worsened, death, and other condition. In the current study, the number of decedents was used as a measure of fatality.

Information regarding the following patient characteristics was collected: age, sex, duration of hospitalization, and receipt of public assistance. The Japanese public assistance program provides social benefit to people who are classified as destitute according to their daily needs.

Regional data were obtained by summing municipal data regarding the number of psychiatric hospital beds per 1000 population in the health region as of 1 October each year (derived from the Survey of Medical Institutions). The total population as of 31 March each year was derived from the Basic Resident Register and Population (Ministry of Internal Affairs and Communications). The prefectural government plans the allocation of healthcare resources based on health regions (“the secondary tier of medical care”), which usually include multiple municipalities. During the study period, the number of included municipalities decreased from 3252 to 1741 because of a municipal merger, in which the boundaries of health regions were revised. Therefore, each variable was calculated based on the boundaries of municipalities and health regions as of 1 October 2014.

The locations of patients’ residences and hospitals were classified into four categories: same municipality, different municipality in same region, different region in same prefecture (state), and different prefecture.

### 2.5. Ethical Considerations

The completion and return of the survey implied consent; therefore, the hospitals were not required to sign consent forms. To preserve respondents’ anonymity, identification numbers were assigned to hospitals and patients. The study was approved by the ethics review board at the Tokyo Metropolitan Institute of Medical Science (Project No. 15-4, approval on 16 March 2015), and the investigations were carried out following the rules of the Declaration of Helsinki of 1975, revised in 2008.

### 2.6. Data Analysis

Patients were divided into groups according to the means via which deliberate self-harm occurred, and age distribution was compared between these groups. The patient sample was divided into three categories using the 25th and 75th percentiles for each regional characteristic. The national number of patients admitted to hospital because of deliberate self-harm and subsequently discharged was estimated by summing the weighting coefficients for the patients with reference to the national number of suicide cases each September. Suicide statistics were derived from vital statistics provided by the Ministry of Health, Labour, and Welfare in Japan. All statistical analyses were conducted using Stata SE for Windows, version 14.0 (StataCorp, College Station, TX, USA). The two-tailed significance level was set at 0.05.

## 3. Results

### 3.1. Patient Characteristics

Of the 13,014 patients admitted to hospital because of deliberate self-harm, 42.2% were men. The patients’ mean age was 43.4 (standard deviation: 21.5) years. In addition, 40% received follow-up outpatient care from the hospital to which they were admitted. More than half of the patients were admitted to hospitals in the municipality in which they resided, and one third resided in a region with fewer psychiatric beds per population (Table 1). Almost half of the patients (*n* = 6015, 46.2%) were discharged within 2 days of admission.

Of the 13,014 patients, 6025 were admitted to hospital because of injury (ICD-10 codes S00–T34). In addition, 5674 were admitted to hospital because of drug poisoning (T36–T50); of these, 5212 had taken other unspecified drugs, medicaments, or biological substances (T50.9), while 358 had taken antiepileptic, sedative-hypnotic, or antiparkinsonism drugs (T41). In addition, 1056 patients were admitted to hospital because of substance poisoning (T51–T65); of these, 592, 217, and 176 had experienced the toxic effects of unspecified substances (T65.9), pesticides (T60), and carbon monoxide (T58), respectively. The remaining 259 patients included 183, 12, 10, and 54 admitted because of other unspecified effects of external causes (T66–T78); certain early complications of trauma (T79); complications of surgical and medical care, not elsewhere classified (T80–T88); and the sequelae of injuries, poisoning, and other consequences of external causes (T90–T98), respectively. Therefore, patients were divided into following three categories: drug poisoning (*n* = 5677; T36–T50 and T96), substance poisoning (*n* = 1060; T51–T65 and T97), and other self-injury (*n* = 6277; S00–T34, T66–T96, and T98); the proportions of decedents in these groups were 1% (*n* = 57), 6.9% (*n* = 73), and 5.4% (*n* = 341), respectively.

**Figure 1:**
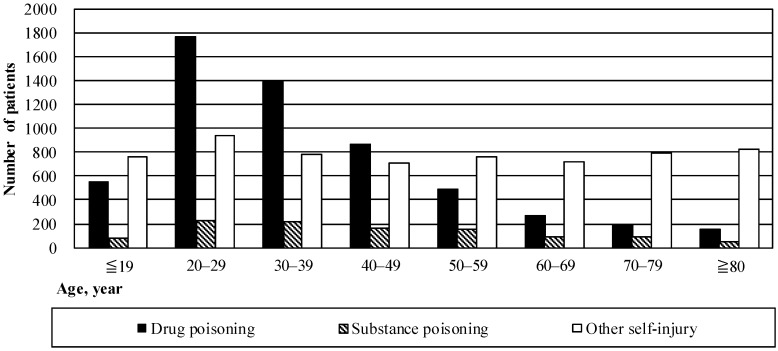
Observed numbers of patients according to age group and type of deliberate self-harm, for every third September between 1996 and 2014.

Patients admitted because of drug poisoning were younger relative to those admitted because of substance poisoning or other self-injury, F(2) = 536.34, *p* < 0.001. Most patients admitted because of drug poisoning were aged between 20 and 39 years. Approximately 800 cases involved other self-injury, across all age groups (Figure 1). 

The graph shows the observed number of patients admitted to hospital because of deliberate self-harm and subsequently discharged, according to age and type of self-harm, for every third September between 1996 and 2014.

### 3.2. Estimation of the Number of Patients Who Engaged in Deliberate Self-Harm

**Figure 2:**
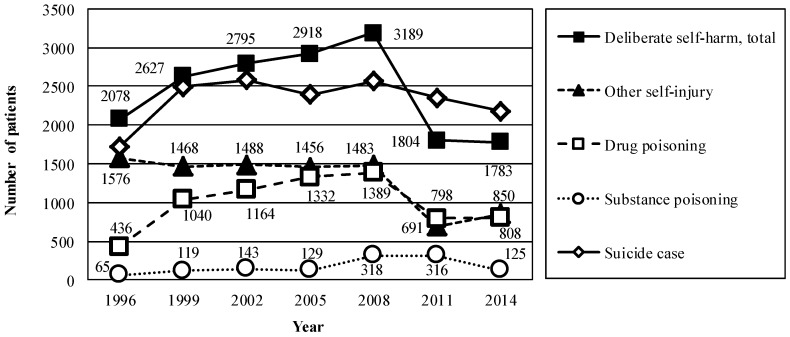
Estimated numbers of patients admitted to hospital because of deliberate self-harm and subsequently discharged, for every third September between 1996 and 2014.

The estimated number of patients who had engaged in deliberate self-harm during the month of September increased from 2078 in 1996 to 3189 in 2008, when the number of suicide cases reached a peak, and decreased to 1783 in 2014 (Figure 2). The estimated number of cases involving drug poisoning increased between 1996 and 2008, and decreased between 2011 and 2014. The estimated number of cases involving other self-injury remained at approximately 1500 between 1996 and 2008, and decreased in 2011. The estimated numbers of cases involving substance poisoning were higher in 2008 and 2011 relative to those observed in the other years included in the study.

The graph shows the estimated numbers of patients admitted to hospital because of deliberate self-harm and subsequently discharged, according to type of self-harm, for every third September between 1996 and 2014. Quarry marks represent the national number of suicide cases for each September. Suicide statistics were derived from vital statistics provided by the Ministry of Health, Labour, and Welfare in Japan.

## 4. Discussion

The estimated number of patients who engaged in deliberate self-harm increased between 1996 and 2008, and decreased in 2011, in parallel with the national number of suicide cases. Approximately half of the patients were admitted because of self-harm involving means other than drug poisoning, which had a relatively high mortality rate (5.6%). This patient group included higher numbers of patients of the male sex and older age, which have been associated with fatal self-harm [2,3].

This was the first study to demonstrate similar time trends for the numbers of suicides and hospital admissions due to deliberate self-harm, using national data. The results provide evidence to support the theory that while there are many differences between suicide and self-harming behavior, some cases of suicide and deliberate self-harm have overlapping spectra [14,16,17]. The results also indicated that the number of patients admitted to hospital because of deliberate self-harm was similar to the number of suicide decedents in Japan. Further, these patients were younger relative to suicide decedents. Whereas younger populations usually have lower suicide rates compared to middle-aged and elderly individuals, suicide prevention should include the former in the target population as well as the older generations. Care for suicide prevention should be provided for those who commit deliberate self-harm, as suggested by previous Swedish research [18].

The proportion of public assistance recipients (8.5%) in the patient sample was higher relative to that observed in the general population (1.67% in 2012) [19]. As economic poverty has been associated with suicidal ideation and behavior [20], receipt of public assistance could be a risk factor for deliberate self-harm. The rate of receipt of public assistance was particularly high in drug poisoning cases. In addition, some recipients of public assistance tend to abuse psychotropic medication [21]. Furthermore, most drug poisoning cases in Japan involved current or previous exposure to psychotropic medication [22]. Deliberate self-harm is also associated with a range of psychiatric difficulties [23,24]. However, recipients of public assistance are excluded from coverage by the public healthcare insurance program, and hence excluded from the regular health-check system that health insurers are obliged to provide in Japan. Therefore, receipt of public assistance could indicate a need for mental health care, and the public assistance program should provide mental health support for recipients of social benefit schemes.

The number of patients admitted because of drug poisoning increased in parallel with the national suicide rate from 1998 onward, when unemployment increased dramatically following the economic recession. Since then, drug poisoning has been one of the most prevalent means of deliberate self-harm. Unlike in the United States, firearms are unavailable to the public in Japan [25]; therefore, drug poisoning is a more accessible means of self-harm. While the Japanese healthcare scheme—which consists of public insurance programs—covers all residents, there is no registration system for general practitioners. Therefore, people can access multiple physicians without mandatory referral or information sharing. Patients who have engaged in deliberate self-harm could be discharged from hospital without referral to their general practitioner. A system via which information regarding deliberate drug poisoning is shared between general practitioners and hospitals should be established.

The estimated number of substance poisoning cases increased in 2008 (318) and 2011 (316). According to vital statistics provided by the Ministry of Health, Labour, and Welfare [26], the number of suicide cases involving substance poisoning (ICD-10 codes: X65–X69) were high in 2005 (*n* = 5062) and 2008 (*n* = 4814). The difference in time trends between cases involving deliberate self-harm and suicide could be attributed to the sampling process used for patients who had engaged in deliberate self-harm. As our sample was limited to patients who had been discharged from hospital, those who had not received inpatient care were excluded. Future studies should use epidemiological designs to capture the national prevalence of deliberate self-harm in Japan.

The present study was the first to estimate the national number of hospital admissions due to deliberate self-harm in Japan. The National Patient Survey did not include ICD-10 codes V01–Y98 (external causes of morbidity and mortality); therefore, information regarding certain means of self-injury, such as hanging, jumping, cutting, or piercing, was unavailable. Patients with self-injury would have included those who were treated for hanging, jumping, cutting, or piercing; however, we could not identify these subgroups because of a lack of information. In addition, the National Patient Survey did not collect detailed information regarding comorbid mental illness or other socio-demographic factors. Further, the cross-sectional design did not allow assessment of multiple admissions over time.

## 5. Conclusions

In conclusion, the trend in deliberate self-harm was synchronous with that in suicide cases over time. Suicide prevention strategies should include care for those who attempt suicide and target younger, as well as middle-aged and elderly, individuals. In addition, the public assistance program should provide mental health support for recipients of social benefit schemes, and a system via which information regarding deliberate drug poisoning is shared between general practitioners and hospitals should be established.

## Figures and Tables

**Table 1:** Patients admitted to hospital because of deliberate self-harm and subsequently discharged.

Variable	Mean (SD) or *n* (%)
**Follow up after discharge**	
Decedents, *n* (%)	471 (3.6)
Outpatient care from the hospital, *n* (%)	5392 (41.4)
Outpatient care from another hospital, *n* (%)	3730 (28.7)
Home healthcare, *n* (%)	35 (0.3)
Other situation at home, unspecified, *n* (%)	1941 (14.9)
Admission to another hospital, *n* (%)	1030 (7.9)
Admission to geriatric intermediate care, *n* (%)	67 (0.5)
Admission to residential facility, *n* (%)	58 (0.4)
Other setting, unspecified, *n* (%)	290 (2.2)
**Year of discharge**	
1996, *n* (%)	1271 (9.8)
1999, *n* (%)	1918 (14.7)
2002, *n* (%)	2043 (15.7)
2005, *n* (%)	2170 (16.7)
2008, *n* (%)	2508 (19.3)
2011, *n* (%)	1562 (12.0)
2014, *n* (%)	1542 (11.8)
Age, year, mean (standard deviation) †	43.4 (21.5)
Sex, male, *n* (%)	5495 (42.2)
Duration of hospitalization, days, mean (standard deviation) ‡	15.7 (69.7)
**Location of patient’s residence and hospital §**	
Same municipality, *n* (%)	6994 (54.2)
Different municipality in same region, *n* (%)	3344 (25.9)
Different region in same prefecture (state), *n* (%)	1904 (14.8)
Different prefecture, *n* (%)	661 (5.1)
**Receipt of public assistance, *n* (%)**	**1112 (8.5)**
**Regional number of psychiatric beds per population, patient residence §**	
<25th percentile, *n* (%)	3864 (29.9)
25–75th percentile, *n* (%)	6588 (51.1)
>75th percentile, *n* (%)	2450 (19.0)

† Three cases were excluded because of missing data regarding age; ‡ 32 cases were excluded because of missing data regarding duration of hospitalization; § 112 cases were excluded because of missing data regarding patient residence.
